# Neural Phenomenon in Musicality: The Interpretation of Dual-Processing Modes in Melodic Perception

**DOI:** 10.3389/fnhum.2022.823325

**Published:** 2022-04-15

**Authors:** Nathazsha Gande

**Affiliations:** Department of A-Levels, HELP University, Kuala Lumpur, Malaysia

**Keywords:** musicality, spontaneous cognition, melody, dual-processing, basal ganglia

## Abstract

The confluence of creativity in music performance finds itself in performance practices and cultural motifs, the communication of the human body along with the instrument it interacts with, and individual performers’ perceptual, motor, and cognitive abilities that contribute to varied musical interpretations of the same piece or melodic line. The musical and artistic execution of a player, as well as the product of this phenomena can become determinant causes in a creative mental state. With advances in neurocognitive measures, the state of one’s artistic intuition and execution has been a growing interest in understanding the creative thought process of human behavior, particularly in improvising artists. This article discusses the implementation on the concurrence of spontaneous (Type-1) and controlled (Type-2) processing modes that may be apparent in the perception of non-improvising artists on how melodic lines are perceived in music performance. Elucidating the cortical-subcortical activity in the dual-process model may extend to non-improvising musicians explored in the paradigm of neural correlates. These interactions may open new possibilities for expanding the repertoire of executive functions, creativity, and the coordinated activity of cortical-subcortical regions that regulate the free flow of artistic ideas and expressive spontaneity in future neuromusical research.

## Introduction

The establishment of dual-processing modes in improvising musicians is used to examine the nature of creative cognition, specifically how the experience of improvisers change the balance between spontaneous (Type-1) and regulated (Type-2) processes.

However, there is relatively little evidence that musicians in the non-improvising domain may be perceived to encounter a spontaneous processing mode during the course of performance. Several studies of spontaneous cognition have revealed large-scale functional networks in creativity, notably in areas of the default mode network (DMN) (Fink et al., 2009a,Fink et al., 2012; Ellamil et al., 2012; Beaty et al., 2014; Benedek et al., 2014). The DMN is composed of the posterior cingulate cortex (PCC), the precuneus, the medial prefrontal cortex (mPFC), and both the inferior parietal lobes on either side (Gusnard and Raichle, 2001). When given an external task, this network’s activity decreases, whereas when not given an external task, its activation increases (Raichle et al., 2001; Buckner et al., 2008). While an artist’s attention is drawn to achieving “more explicit and deliberate response” requirements during a timed audition or an examination, the necessity for controlled processing may increase significantly, resulting in the “suppression of default processing” (Mok, 2012). Since the DMN’s discovery, considerable research has been undertaken to elucidate its fundamental function, which may serve as a neural basis for the dual-process model (Gronchi and Giovannelli, 2018).

To evaluate creativity, the body of research in this field has employed “standardized psychometric tests or laboratory-based divergent or convergent thinking activities” (Rosen et al., 2020). While certain tasks are useful for disentangling elements of creative cognition, their ecological validity and domain specificity are limited (Zeng et al., 2011). Alternative methodologies that employ complicated, realistic problems may better depict creative cognition as it occurs in real-world circumstances (Boccia et al., 2015). Jazz improvisation has been adopted by researchers for this purpose (Limb and Braun, 2008; Beaty, 2015; Loui, 2018). This is because the intricacy and temporal constraints of competent real-time improvisation should restrict dependence on conscious, effortful, Type-2 executive processing. In this regard, improvisation is an appropriate test-case for executive-controlled processing (Norgaard et al., 2013; Rosen et al., 2017). Previous studies show that higher Type-1 processes and default-mode network activity promote jazz improvisation (Beaty, 2015; Rosen et al., 2017). However, other studies indicate that experienced jazz improvisation is related to a distinctive brain-state defined by “reduced Type-2 executive control” (Limb and Braun, 2008; Pinho et al., 2014) that suggests that spontaneous and controlled processing may co-occur (Mok, 2014) in creative performance. As these findings of dual-processing modes may be indicative of the fact that improvising artists have experienced a fluctuating sense of self in the real-time execution of improvisation, this concept of “flow” in non-improvising artists may be induced by the thought of the sound they intend to create when it comes to melodic phrasing. They may employ performance parameters and timbral intents (i.e., tone colors) to generate variability in musical interpretations of a written work beyond the known common interpretations played previously.

The aim of this article is to illustrate how these findings of a dual-process model may be integrated within musicians that perform a work through-composed, pre-determined written music and how this influences the way melodic lines are conceptualized. As a new area of study, it draws on the philosophical and interpersonal research approaches established in the social sciences as well as the modern neuropsychological methods developed in the natural sciences. It implies a multimodal musical conscience as opposed to the understanding of music as a pure auditory phenomenon.

## Neural Implementation of Musical Creativity

Previous study on artistic creativity suggests that the frontal brain regions may play a unique purpose in the creative thought process ([Bibr B63][Bibr B47]). In the same frame of mind, (Arban, 1864) the creator of the Arban Method of a complete pedagogical method for students of trumpet, cornet, and brass instruments stated: “above all things, endeavor to hear good music well interpreted…seek out amid singers and instrumentalists, the most illustrious models, and this practice having purified their taste, developed their sentiments, and brought them as near as possible to the beautiful, may perhaps reveal to them the innate spark which may someday be destined to illumine their talent.” This example highlights the complex process underlying the generation of a creative idea in performing artists during the time course of creative thinking. Although several cognitive functions are required during the temporal evolution of creative thinking, research agrees on the interdependence of generative capacities and evaluative abilities in the emergence of creative ideas (Mumford et al., 2002; Beaty et al., 2016). Despite a growing number of studies highlighting the significance of divergent thinking (DT), we still know relatively little about how the brain stimulates creative cognition— a cognitive process used to develop numerous potential original solutions to an open-ended problem (Guilford, 1959; Barbot and Lubart, 2012). Musical identification and brain processing are only a few of the applications which can have cognitive and emotional implications as this extended chain of neuronal processes are affected by several brain areas and functions (central and peripheral). The task of then executing musical improvisation as a specific attributive human quality in music performance induces a creative mental state particularly analyzed in improvising musicians (Keller, 2014; Lopata, 2014; Toropova et al., 2016; Sattin et al., 2021).

Fink and Benedek (2012) examined EEG research which particularly centered on the relationship between creative ideation (i.e., divergent thinking) and control within the alpha recurrence band (8–12 Hz). Their investigation uncovered compelling evidence that EEG alpha power is particularly responsive to a variety of creativity-related demands: alpha power changes according to the needs of creative tasks (the greater the degree of alpha, the more creative a task), as a function of originality (more originality is coupled by more alpha), and as a measure of a participant’s creative level (more alpha found in higher creative individuals, see Fink and Benedek, 2012; Fink and Benedek, 2013). Based on these findings, Fink and Benedek (2012) determined that the recorded alpha analyses are among the foremost reliable findings within the neuroscientific research of creativity. As a result, we may conclude that studying alpha power variations is a significant and reliable tool for studying brain activity patterns throughout the process of the creative idea development. The increments in EEG alpha modulation (hereinafter alluded to as alpha synchronization) have traditionally been viewed as an indication of “cortical idling” (Pfurtscheller et al., 1996).

According to past research on creativity, alpha synchronization may signify a situation of high internal processing demands defined by “the lack of bottom-up processing” (Ray and Cole, 1985; Cooper et al., 2003) and in this way, it can be classified as an immaculate frame of “top-down activity” (von Stein and Sarnthein, 2000, p.311). This top-down mechanism may have an attentional control role, which results in the blocking of task-irrelevant inputs (Klimesch et al., 2007). In this manner, alpha synchronization is most likely linked to selective and active cognitive processing (Sauseng et al., 2005), which has been consistently detected over the prefrontal and temporo-parietal cortex during creative ideation (Fink and Benedek, 2012), and gives rise to active cognitive processing and focused mental attention (Fink et al., 2009a; Benedek et al., 2011; Fink and Benedek, 2012; Jauk et al., 2013). Previous research has revealed that highly creative individuals had better alpha synchronization at right parietal regions than less creative people (e.g., Martindale and Hines, 1975; Jaušovec, 2000; Razumnikova, 2007; Fink et al., 2009a,b). Bhattacharya and Petsche (2002, 2005) and Petsche et al. (1997) discovered frontal alpha desynchronization in studies involving the visual stimulation of artistic tasks, whereas Fink et al. (2009b) discovered alpha synchronization in a study involving the mental imagery of spontaneous composition of jazz improvisation. Fink et al. (2009b) findings did not follow the previous studies’ pattern, although it may be argued that their task had better ecological validity, and that activities involving visual arts activate frontal brain areas for distinct or extra processes than non-visual arts tasks (Dietrich and Kanso, 2010). Overall, the EEG research yielded no compelling evidence of hemisphere specialization during artistic thinking. Several studies, however, reported greater alpha power in the right hemisphere (Martindale et al., 1984; Fink et al., 2009b), along with increased right hemispheric synchrony in other frequency bands (Petsche et al., 1997; Bhattacharya and Petsche, 2002, 2005).

## A Dual-Process Model: Implicit and Explicit Processing

It appears that these studies support the hypothesis that improvisation relies on the interaction between new motor sequences of “generative, executive, and evaluative processes” on performance monitoring, the facilitation of attentive processes toward higher-order objectives and the minimization of elaboration requirements (Pressing, 1987). Despite the fact that this explanation acknowledges the existence of some ecological complexity in improvisation, not much have been mentioned about the subcortical mechanisms that underlie the occurrences in question. When it comes to an integration of emotional, cognitive, and motor information, two separate systems are needed to achieve it (Dienes and Perner, 1999). One is explicit, based on rules and content, linked to the superior cognitive functions of the frontal and prefrontal lobes, and the medial temporal lobe; the other is implicit, more efficient and based on practical, unconscious skills, linked to the supporting abilities, particularly in the basal ganglia (Schacter, 1987).

The explicit system facilitates a hierarchical elaboration of information in this representation, with the bulk of the most advanced mental abilities being dependent on the construction of a higher order: the prefrontal cortex. Naturally, there is no tight distinction between these systems and the neurological structures upon which those cognitive abilities rely (Oliverio, 2008). Indeed, both systems may be active concurrently, and the striatum, a key component of the basal ganglia, participates in explicit cognitive activities *via* complicated connections to the prefrontal cortex (Graybiel, 1997; Cotterill, 2001). Additionally, the striatum integrates data from diverse cortical regions that are converging on their respective terminal fields (De Leonibus et al., 2005). The evidence for the ventral striatum’s function demonstrates that the accumbens not only plays a critical role in either positively or negatively reinforced behavior, but also serves as a critical node in the network of emotive elaboration of information from the amygdala, midbrain nuclei, hippocampus, and prefrontal cortex (Mele et al., 2004). This interconnected system is critical because it facilitates the elaboration and translation of information into appropriate behavior that may be reinforced. Additionally, the ventral striatum predicts the pleasure of decisions and alerts to the negative consequences of a reward-seeking activity (Cotterill, 2001). The existence of reward anticipation has been demonstrated in reward systems. Similarly, the starting point of a predetermined melodic work—for example, a melodic fragment or the beginning of a musical phrase—creates an anticipation of completion owing to the connection between the parts and the perception of a piece of music (Boulez et al., 2014).

Observations in novel or unexpected circumstances may stimulate the basal ganglia, confirming the validity of prefrontal cortex-formulated rules (Rowe et al., 2008). Dissonances or differences between perception and anticipation are likely to activate chemical signals in the basal ganglia, which is a plausible conclusion. Furthermore, the ventral striatum intervenes in cognitive methods to adjust to environmental demands according to immediate requirements. As a result of their interactions with the frontal cortex, limbic and striatal systems, the subcortical mechanisms of reinforcement play an important role in the processes of decision-making, judgment planning, and selecting appropriate action (Bechara et al., 1998).

For example, it is well recognized that primary thought processes, such as free associations, mind wandering and daydreaming, function at a low degree of activation, whereas secondary thought processes (characterized by abstract, logic, and reality-oriented cognition) require attention and operate at a higher level of activation (Oliverio, 2008). Activation of the prefrontal cortex has been linked to an increase in “target-oriented” conduct by reducing “irrelevant” mental action and connections (Oken et al., 2006). On the continuum of cognition between primary and secondary processes, creative people are more likely to bounce between these two levels of thinking, going through a primary state of awareness that facilitates novel combinations of parts (Fromm, 1978). A solution (sometimes misunderstood as creative activity) is found by transforming secondary processes into primary processes, enabling free connections to occur. The ability to “switch off” the prefrontal cortex and convert secondary activities into main processes is linked to the quest for new answers (Sternberg, 1999).

Csikszentmihalyi (1996) used the word “flow” to characterize a state of exuberant attention associated with decreased prefrontal activity. In this case, the attention of the artist seem almost natural, effortless, and focus is so intense that that one becomes completely absorbed in the material and ignores everything else. As a result, implicit cognitive processes are likely to activate, enabling the user to utilize their inherent abilities and cognitive functions without requiring external assistance (Dietrich, 2004). To put it another way, the analytical ability of the explicit system is briefly “turned off” as a transient condition of low-level activity in the prefrontal lobe. According to the previous research, the focus on a specific objective appears to contradict evidence of limited frontal brain activity. As a matter of fact, due to its unbroken nature, the flow necessitates the activation of the frontal attentive net (Ulrich et al., 2014). However, in altered states of consciousness, the ability to concentrate one’s attention is also present by way of transient hypofrontality (Posner, 2004). The reduction in prefrontal function that occurs when someone is in a state of flow also helps to alleviate the symptoms of self-consciousness (Rubia et al., 1999). These and other considerations lead most scientists to classify the flow state as a lower one than executive attention, which allows the mind to concentrate on a single object while “shutting off” the prefrontal cortex’s higher-order processing functions (Dietrich, 2003). By concentrating on the current action, the implicit system is able to operate at peak efficiency. The prefrontal cortex takes them in, but they are returned to the basal ganglia as soon as they become repetitive practices, to be changed into an implicit process (Baìez-Mendoza and Schultz, 2013). Using this approach, we may claim that the prefrontal cortex (with its dorsolateral regions) acts as a process that continually creates new knowledge into creative behavior, while the basal ganglia (and its implicit strategies and memories) function as a mechanism for new information acquisition.

A single structure or system, on the other hand, cannot adequately describe the complexity of cognitive functions (Tsukada and Agranoff, 1980). Language is formed not just by the left motor and sensory centers, but also by the nets that link these regions to the basal ganglia (Chakravarthy et al., 2010). Given that creativity can be understood in terms of its neural and cognitive correlates, including implicit and explicit strategies, “primary and secondary states of mind, executive ability, scope-oriented behavior, and emotionality” (Jung et al., 2010), it is also true that the plastic processes that enable environmental adaptation *via* novel and original strategies play a lesser role in this context (Oliverio, 2008).

## Existing Implementation of a Dual-Process Model in Real-Time Music Performance

During the creative process, improvising artists experience a fluctuating sense of self. According to De Smedt et al. (2016), while performers seem to be more innovative while improvising, many musicians report a “flow” in which they are deeply absorbed in their performance. “Yielding,” “being present, and not being present” are all terms used by Nardone (1997) to characterize improvisation as a binary condition (pp. 127–128) that may mirror a dual-processing mode.

In recent studies, several researchers (Rosen et al., 2020) assessed conflicting theories using jazz guitarists (*n* = 32) to identify creativity whether it is “primarily based on automatic, unconscious (Type-1) or controlled, conscious (Type-2) cognitive processes” that can contribute to creative thought (e.g., Nijstad et al., 2010; Sowden et al., 2015). The combination of these types of cognitive processes is dictated by individual differences (e.g., competence and personality) and circumstances (e.g., instructions) using high-density electroencephalograms (EEGs) to examine the neural correlates of jazz guitarists with varying levels of creative quality and domain experience during creative output. Similarly, (Rosen et al., 2017) had jazz pianists improvise to a new chord sequence and rhythm accompaniment. Among the many skills required to successfully improvise are the ability to quickly switch between chords, note choices, and rhythmic execution. During instruction-activated type-2 processes, novice improvisers refocus their attention on an objective of creative expression, recruiting strategies that are likely to produce an innovative product, and avoiding “cognitive fixation” (Howard-Jones, 2002) that can occur because of “limited domain knowledge and proficiency” (Green et al., 2015). While expert improvisers’ creative output was unaffected by the increased use of Type-2 controlled processes, they rely more largely on Type-1, implicit processes. Increased domain-related functional connectivity is shown in experienced improvisers (Pinho et al., 2014), indicating that their extensive training and experience favors Type-1 processes or an adequate balance of Type-1 and Type-2 processes (Mok, 2014; Pinho et al., 2014). As a result, no difference in the quality of expert improvisations was found when a group of experts used creative instructions to activate extra Type-2 processing (Rosen et al., 2017).

It was also shown that the perceived quality of improvisations rose when the musicians reported feeling more in the flow after improvising. This suggests a relationship between perceived quality of jazz improvisations and the phenomenological experience of obtaining a flow-state during improvisation performance. The negative relationship between high expectation chord evaluations and improvisatory training implies that it inhibits sounds that are too predictable or routine and encourages a higher tolerance, or relative preference, for more unforeseen or complex stimuli. This may be the case as improvising artists may have agreed to participate in jazz improvisation training in the first place due to their affinity for innovation, unexpectedness, and individuality.

## Dual-Processing in Melodic Perception

While expanding musical-harmonic knowledge may not be a priority when playing pre-determined melodies in written music, non-improvising artists may concentrate on the deliberate sounds they desire to create for the sound of their art. This is consistent with Berlyne’s (1971) hypothesis on the link between desire and growing complexity, which states that for persons with greater domain-specific expertise in the subject, the ideal complexity changes toward being more complicated for people with more domain-specific knowledge within the field (Berlyne, 1971). This may be the case when non-improvising artists want to explore other ways pre-determined written music is performed. Furthermore, an artist who performs a unique interpretation of a certain work may be considered creative and innovative since the artist deviates from the usual conventional interpretation done in the past.

When non-improvising musicians were asked to improvise, it was observed that the increase in executive area activity found in classically trained musicians was most likely related to the greater metabolism required by the improvisatory task, owing to the players’ lack of familiarity with improvisation (Maldonato et al., 2017). Pinho et al. (2014) reported that expert performers have less activation of the lateral prefrontal cortex than non-expert performers, but they also report an increase in functional connectivity in prefrontal areas, which may reflect a greater automatism in executive processes (de Manzano and Ulleìn, 2012). Another explanation is that decreased activity of certain regions in experienced musicians reflects increased neural efficiency (Grabner et al., 2006) as improvisation would necessitate simultaneity in the execution of diverse cognitive and motor tasks and sequences. In comparison to the inexperienced musician, the skilled musician is better equipped to deal with such processes, which may explain the decreased metabolic activity in the lateral prefrontal cortex (Neubauer and Fink, 2009). Thus, increased functional connectivity among the numerous areas involved in those processes may indicate increased neural efficiency as a result of practical experience: if neural connections were more gradually consolidated in non-improvising musicians, this favors a more efficient information exchange among the structures of a larger net.

Naturally, further methodological discrepancies might be identified at the root of the imbalance, exhibited in the models described in the specialist literature. For example, Limb and Braun (2008) allowed musicians unlimited expressive freedom (in terms of harmony, melody, and rhythm), which clearly resulted in changes in the metabolic activity of several additional brain regions. If the experimental conditions were similar to those encountered in real life, it follows that individualization of neural circuits involved in improvisation is more challenging (Berkowitz, 2010). These findings, however, may differ as non-improvising musicians place a value on the purposeful quality of sounds they aim to achieve when performing a piece of music, rather than extending musical-harmonic vocabulary as a mark of their musical craftsmanship.

## The Art of Phrasing Melodic Lines:

### Appreciation of Tone Color

There have been studies on single-tone pitch perception that usually suggests a predominance of right-hemispheric brain networks, but only in certain areas of pitch processing (Zatorre et al., 1999). Because of this, basic frequency discrimination is either minimally or not at all impaired by unilateral cortical lesions (Milner, 1962; Zatorre, 1988) and may possibly be completed *via* subcortical structures. The Mismatch Negativity (MMN) mechanism is a specific dimension of music listening and is used to measure tone component variations within auditory Event Related Potential (ERP).

Previous studies on Mismatch Negativity (MMN) have established correlations between a variety of sound characteristics, including pitch, tone, source location, but also dynamics and rhythm (Xide et al., 2015). The research on MMN response spans a broad range of disciplines, from simple sound detection to complicated music perception. Researchers (Koelsch et al., 1999) found that musicians (violinists) knew around 80% of the changed chords (tones played simultaneously), and non-musicians knew about just 10% of them. To determine chord intervals, it may be required or beneficial to concentrate on tone constituents. This approach appears to be open to musicians, and some of them, particularly the most accomplished, might utilize a similar technique even when the objective at hand is only recognition. It shows that short sound sequences are established by this element of the melodic structure. The ability to recognize melodies relies on the ability to recognize the contours of a tune (Dowling and Fujitani, 1971). Because of this, musicians may be more sensitive to the MMN than non-musicians to input changes. Based on the aforementioned finding, it is possible that trained musicians are particularly adept in discriminating between tones within a single tonal series.

Researchers found that MMN enhanced amplitude was induced by earlier music education and not by earlier variations between groups (Putkinen et al., 2014). As a consequence, it is possible that the development of sensitivity to diverse functions of sound (time, dynamics, tone, interval, contour) is not uniform, but derives from long-term schooling within a particular music genre. It might activate the MMN-described mechanisms, which could lead to EEG-recorded alterations in the neural generators of musical and non-musical groups (Tervaniemi et al., 1997).

However, within the non-improvising domain, musicians will establish a greater connection to the music conveyed if artists adjust their expectations from “pitch-precision and melodic flow” to a specific understanding of tone color (Bellando and Deschenes, 2021). Ortmann (1935) used the concept of “tone quality” and proposed that it is subjective and derives from our collective reaction to three variants: pitch, intensity, and duration. These may involve higher-order processes (perhaps involving frontal-lobe structures) and lower-order systems (primary cortex) to interact so that only the most salient stimulus elements are picked for further processing of a specific tone quality of pitch. Bernays and Traube (2014) proposed the term “composite timbre” to describe the interplay between instrumental timbre and other performance parameters when discussing timbre in a musical and polyphonic context. Musicians were asked to create and perform pieces of music based on five timbral characteristics: dry (without color), bright (with color), round, velvety and dark. Creating “explicit timbres in their performances” is important from the performers’ perspective because it leads to self-satisfaction and a sense of accomplishment (Holmes, 2011), helps artists perceive expressive elements more holistically (Juslin, 2000), or be relevant to emotional expression (Li and Timmers, 2020) which would encourage variability in execution. It is a conscious physiological process for non-improvising artists to recognize and differentiate between the sounds that have significance, and then analyze those that do not (Zwoliṅska et al., 2019). The receipt of sensations, the structuring of perception, and the identification of sounds are the fundamental cognitive requirements for understanding the auditory realm.

It was found that pianists use timbral intentions extensively in their piano performances, and the results show that a pianist’s understanding of timbre is enhanced by an embodied experience, such as bodily and mental awareness; suggesting the importance of mental preparation in piano performance (Li and Timmers, 2020). The tone color of each player’s sound is customized by the performer by adjusting variables such as air volume and pressure, as well as the embouchure: the form of “lips, mouth, and throat, heart rate, muscular tension in the upper body and diaphragm, posture” and so on. Many of these variables are not under the player’s conscious control, which may be due to spontaneous processing. Thus, their interaction with the instrument yields a tone-coloring sound that emulates the player’s unconscious state, a proposition that is somewhat innate for experienced musicians, as they may be familiar with the piece of music being performed. Bellando and Deschenes (2021) claims that the performer strives for: an “as is” portrayal of their mental and physical condition, expressed without the player’s artificial effort. Many adherents of this philosophy prefer the “honest” tone-color to the anticipated pitch, as the former cognitive processes may be primarily based on “controlled, conscious (Type-2)” effort on the part of the musician, whilst the latter is determined by the “automatic, unconscious (Type-1)” process on the state of the artist’s mind and body. Furthermore, to better communicate timbre-related performance goals, music educators and students alike often use “metaphors, gestures, and modeling” to emphasize their intended effects (Li and Timmers, 2021). However, it is uncertain whether students can effectively express timbral intentions to listeners in a range of auditory-visual presentation circumstances since they may be more focused on the technical aspects of the written music, such as pitch, timing, and rhythm, all of which may be influenced by controlled processing. This may be the case as novice artists may not prioritize the aspect of communicating the melodic lines artistically. Instead, they may rely on imitating a traditional interpretation of the work, acquiring “controlled, conscious Type-2 processes” with the primary emphasis on what they think “should” be heard rather than enhancing their performance communication, which is something that expert musicians do spontaneously. In this sense, non-improvising artists focus on the particular sounds in melodic phrasing that requires recognizing new timbres, comparing them to previously heard ones, classifying them as significant or not, and making educated guesses about how the sound will improve in the future. It is the brain’s own background and experiences that provide meaning to music while it is being listened to, rather than the music itself. As a result, music comprehension might be either subjective or objective (providing components allowing for a common impression).

### Performance Parameters

The development of musical thought is derived from musical analysis and the skeletal structure of a piece to help guide ideas on phrasing: note grouping, breathing, vibrato, rubato, dynamics, and articulation are some of the components needed to keep and breathe musical communication to audiences.

A plethora of studies have been undertaken to investigate artistic music performance of predetermined melodic lines through studying the general performance parameters (as opposed to musical parameters such as pitch, harmony, or rhythm) that musicians use as expressive devices (Rink et al., 2011). By far the most studied expressive control parameters have been “timing and amplitude,” due to their prominence among the performance parameters that instrumentalists use to vary their interpretation when performing a piece (Bhatara et al., 2011), as well as the relative ease with which they can be measured (e.g., with MIDI digital recording pianos or with acoustical analysis Goebl et al., 2008). Research on “expressive timing deviations from a non-expressive metronomic version” has been ongoing for a long time, to which these timing deviations comprise an “individual expressive microstructure” (for an overview, see Clarke, 1995). No inferences can be drawn about the expressiveness or originality of these performances by studying the lengthening of note values in a final ritard for numerous separate performers or recordings from the twentieth century (Wöllner, 2013). As a result, classical music emphasizes on subtle timing perturbations and variations in dynamic intensity that contribute to the different interpretations of the same melodic line. A musician’s perception of tone can benefit from familiarizing themselves with timbre and other performance parameters (e.g., intensity, articulation, and pace) to induce variability in execution. When performing a piece of music, sudden lapses or variations in intensity that do not comply with the conventional expectations may generate “surprise and other emotional reactions” (Huron, 2006) and reflect the individual performer’s musical intentions.

However, within the limits imposed by these structural constraints of time and dynamics, there is still ample room for musicians to express themselves uniquely when interpreting a work, and whether the dual-process model influences how creatively non-improvising artists can think about the sound (i.e., tone color) they intend to create and shape the written sequence of notes to bring out expression using performance parameters. In addition, non-improvising experts and novices may engage differently in Type-1 and Type-2 processes during the creative production of performing a work through-composed, pre-determined written music. The basal ganglia may be involved in some sort of “dialogue” causing this (Maldonato et al., 2017). Motivation, cognitive and emotional information is processed by the thalamus and other brain areas in order to create abstract patterns and concepts from sensory-motor and explorative experiences (Penfield, 1975). The prefrontal cortex, temporal lobes, and the cingulate gyrus, as well as the orbitofrontal regions of the frontal cortex, all play a role in the development of subjective preferences for reward and pleasure (the lateral parts of the latter influence the choices made depending on the context, resulting in originality, coherence, and so on), all of which go hand in hand with higher levels of decision-making (Koechlin et al., 2003). Azuar et al. (2014) suggests that the task of the performer is then filled with potential outcomes, in this case, the different musical interpretations of the same melodic work.

However, one might argue for these contradictory findings, as there may be a difference in the types of creativity measured, with some requiring prefrontal activation and others not (Dietrich and Kanso, 2010) or experiencing a creative mental state (Lopata, 2014).

### Different Interpretations of the Same Piece

During the course of a performance, expert artists of similar caliber may execute musical phrases without having to emulate someone else’s ideas. They can expressively create interpretive ideas for themselves and execute passages with technical command that fulfill the composer’s intention while still being able to satisfy their own innate musical sense. Although these mental processes of expert artists may encourage Type-1 processes or a sufficient balance of Type-1 and Type-2 processes, here, we explore that the primary focus both artists have is on the purposeful sounds and performance parameters they plan to generate when performing composed, pre-determined written music.

This is shown in the works of Benjamin Britten, a British composer of the Twentieth Century whose work has won the admiration of both novices and seasoned musical audiences. Britten’s compositions are highly regarded because he writes in a distinctive tonal language that “pushes the boundaries of functional tonality,” which is commonly apparent in his solo oboe compositions (Djiovanis, 2005). While Britten’s expressive markings are rather detailed and precise, his musical intentions are described in terms of how different artists perceive and perform them. Several musical interpretations of Britten’s work have succeeded in portraying his musical intentions. Examples include Marilyn Zupnik’s rendition of Ovid’s Six Metamorphoses on her compact album “Classis Oboe Etudes” (Britten, 1998), which confides to be an exceptional depiction of Benjamin Britten’s goals for the sound of his music. As a member of the Israel Philharmonic Orchestra, Zupnik precisely follows Britten’s ideas and markings; the work then sounds as natural as the composer intended.

In the last movement of the Six Metamorphoses after Ovid, “Arethusa,” she closely follows Britten’s emotive marks by employing vibratos in this passage that is controlled and delicate but takes some liberties with pace and pulse as a mark of her musical skill and craftsmanship. In this sense, Zupnik’s “liberties are appropriate” and “never jeopardize Britten’s objectives” (Djiovanis, 2005). She explores this sense of autonomy in measures 5 and 12 but does something unusual and successful in her interpretation of “Arethusa”. In these measures, the performer may be heard dropping to a piano dynamic that is not indicated. The result, however, is that the written crescendos in these measures are intensified. This is an example of Zupnik’s emancipation of the composer’s work as an artistic endeavor for her interpretation. In this respect, Zupnik is allowed to indulge in liberties that are appropriate and never jeopardize the composer’s original intentions.

In the same movement, John Mack, the retiring principal oboist of the Cleveland Orchestra, closely follows Britten’s expressive marks and tempo markings in the sixth movement, “Arethusa” (Britten, 1985). He matures his sound by juxtaposing the “percussive and legato” passages in terms of tone color. It should also be noted that he is meticulous and precise when it comes to playing the pauses in the last eight measures. Because of this, he manages to make the monotonous voice of the oboe sound brilliant while making many of its bright tones sound opaque. In his beautiful interpretation, the movement becomes more fluid and somber. In terms of structural as well as more esoteric musical features, John Mack’s interpretation of Britten’s “Six Metamorphoses after Ovid” is played in a manner that closely resembles the original composition. In certain cases, Djiovanis (2005) suggests that the recording is not as technically precise as Marilyn Zupnik’s recording, but it appears to have “a human and multidimensional aspect about it that many other recordings lack.” When it comes to artistry and how a piece of music is performed, this “gain” in a “multidimensional quality” pertains to how each musician explores their instrument’s tone spectrum and how it leads to a feeling of self-satisfaction and accomplishment for the artist (Holmes, 2011). As far as it may be apparent to improvising artists to experience a neural phenomenon during real-time improvisation, could this extend to several artists’ interpretations of the same piece? It was evident in Britten’s work “Six Metamorphoses after Ovid” that two musicians of similar caliber playing the same movement delivered the piece differently, with one considered to be “better” in the sense that it had fulfilled this “multidimensional quality” in executing the written material.

The musical material may be defined as a single unit that perceives sound occurrences with comparable physical qualities over a specific period of time through dual processes. Insofar, it unifies and integrates discrete temporal events, this process appears to be critical for the experience of music performance. With new sensory experiences, the mind compares past memories, foreshadowing, and expectation (Maldonato, 2014). Consequently, musicians’ enhanced ability to distinguish between different types of stimuli is a result of both higher cognitive and pre-attuned memory-based processing (Koelsch et al., 1999). This mechanism appears to be crucial to the perception of time in that it unifies and integrates separate temporal occurrences. The basic role of memory in artists performing a written piece of music is therefore redefined by the recollection of phrases, performance parameters, and musical styles, within the movable constraints between current occurrences and future expectations. Instead of looking back at the past, a performer’s mind is always scanning forward into the future, such as pre-musical centers of experience, pictures, and atmospheres that are always changing, and open to infinity of advancements and oblivion (Cappelletti, 2016).

Furthermore, it is crucial to establish the neuroscientific basis for a creative state of mind in the non-improvising domain to truly understand the underlying processes that occur when the artist performs a written piece of music. As an outcome, we must devise new methods for integrating an artist’s perception that coincides with a neural phenomenon.

## Creative Mental States in the Performance of Predetermined Written Melodies

In neuroscientific studies on creativity, theoretical frameworks have been postulated to characterize creative cognition and its many phases. The degree of phases strongly implies that the creative process varies (e.g., Wallas, 1926; Finke et al., 1992; Csikszentmihalyi, 1996). In the “Gene-plore” paradigm, for example, two phases are theorized (Finke et al., 1992). As per this model, creative cognition is the product of circuit processing that includes recurring, generating, and exploratory stages (Finke et al., 1992). The authors claim that idea generation begins with the formation of mental representations (the generating phase), and then proceeds through interpretation and modification processes (exploratory phase). Several models offer four (preparation, incubation, illumination, and verification; Wallas, 1926) or even five phases (preparation, incubation, insight, evaluation, and elaboration; Csikszentmihalyi, 1996).

Given the general neglect of phenomenology in music performance studies, a small body of phenomenological works has gathered (O’Cluánain, 1979, 1981, 1987; Giorgi, 1984; Melrose, 1989; Conrad, 1990; Reinders, 1992; Nardone, 1996; Bindeman, 1988). Though they claim to be “phenomenological,” many of these studies use the term in an undefined manner, resulting in qualitative work that Kvale (1994) describes as a “boring empiricist collection of interview quotations, rather than a well-told, convincing tale.” In this regard, additional phenomenological study is needed to understand the nature of musical creativity. One of the more rigorous phenomenological investigations will be briefly discussed, as it is research that adheres to phenomenological psychology’s attitude as well as methodology. According to Reinders (1992), open-ended interviews with artists from a variety of creative disciplines were conducted to better understand artistic innovation in three disciplines: choreography, painting, and musical composition. It appears that certain parts of the artist’s “life-world” are viewed from an aesthetic perspective. An overwhelming sensation of “lack” is created, which drives this artistic need to produce an artwork that will “speak” to this sense of lack. These activities resemble the creative processes described by improvisational artists (Berliner, 1994; Nardone, 1997; Berkowitz and Ansari, 2008), and an intuitive mental state and the fluctuation between intuitive and analytical states have also been particularly characterized by improvising artists (Nelson and Rawlings, 2007).

It was deemed that expert musicians have an intuitive understanding of a musical motif in the direction in which they should conduct their artistic quest to satisfy their sense of artistic need. An explorative phase takes place between the artist’s desire to fill a perceived void and his intuited creative aim (Melrose, 1989). The artist’s intention is also clarified through this experimental approach of practice and tone-coloring to certain phrases he intends to produce. According to Reinders (1992), the experimental inquiry is directed by what he calls “the needs of the aesthetic object.” In this case, it is musical phrases or “units” that bring the work closer to realizing the intuited purposeful object. These are recognized by the artist throughout this experimental process. When it comes to the creative process, Nelson and Rawlings (2007) reported on artists’ descriptions in which they expressed what it was like to be a witness to a more intuitive artistic voice or vision. The artist believes that the spontaneous finding of satisfying artistic structures is beyond his or her control in which activities are guided by their creative intuition of the intended object and are receptive as they remain open to new artistic possibilities.

While engrossed in a creative endeavor, musicians may perceive a “movement” between intuitive and analytic modes of interaction, according to Nelson and Rawlings (2007). A sense of profound connection (or merging) with the work was felt by the artists when they were working in the intuitive mode, but they were disengaged when working in the analytical mode. Intuition and an innate feeling of what feels correct are more important to the creative person than analysis or critique. To put it another way, in the creative zone, one suspends logic and analytical thinking in favor of an intuitive mental state. For example, focusing on the density and tone color of a single note, the closing of a phrase, or returning to the last place in the artwork where a sense of ease with the activity was felt. When the artist engages in a more technical aspect of the artwork, the artist strives to engage an analytic mode in some way (e.g., emphasis on articulation, bowing, dynamics, alternate fingerings) or by working on a new project that requires a more analytical approach (e.g., rhythm, harmonic analysis, etc.).

Consequently, novice artists, such as student musicians or undergraduate music students, may be deprived of artistic knowledge due to a lack of previous artistic experience and may differ from trusting their own artistic intuition and perception, relying more on a mentor’s interpretation of a particular piece or phrase. Reinders (1992) proposes the causation of another work paradox as “distant-engagement,” an alternate between immersing oneself in material manipulation and withdrawing oneself or viewing developing creative forms from the standpoint of the audience. Central to Reinders’ account, novice musicians may tend to withdraw themselves from the idea of creativity when priority is on the technical assurance of the repertoire being studied or if one can think about the music artistically but can’t physically execute it with panache due to normal psychological reaction, new environmental stimulus, technical ability, or the alternate of immersing oneself in material manipulation. Lopata’s (2014) claim underlines this as “a lack of immersion in the spontaneous processing mode.” This may suggest that novice musicians may develop a preference for digesting musical ideas in a systematic mode as they gain expertise. However, as their musical lexicon and theoretical understanding expand, they may approach performance from an analytical, logical, and rational processing style.

## Implications for Future Research and Practice

As a byproduct of phenomenological studies and recent studies of dual-process contributions in real-time jazz improvisation, there is certainly an intersection of common themes between artists in the creative sector: responding to the artwork’s demands, such as not feeling in control of the process; relying on intuition and emotional processes; the sense of discovery through interaction with a medium (the music score or instrument); and the interfering role of conscious, critical, and mental processes. For musicians in the non-improvising domain, further research is needed to support the dual-process model in the paradigm of neural correlates for the execution of varied musical interpretations. Artistic musicality may be approached in a novel way that sheds light on its function in human brain development as non-improvising artists may experience the fluctuating sense of Type-1 and Type-2 processes or an ideal balance between dual-processing modes (Mok, 2014) during creative performance. In addition, computational models used in the study of physiological and pathological music processing may be a valuable supplement to the research on the genesis of predetermined melodic performance.

The description of a phenomenon in independence from issues of connection, explanation, and prediction is not only impossible but also undesirable. However, an in-depth understanding of how a phenomenon manifests itself might naturally lead to thoughts regarding its relationship to other events. Thus, phenomenological inquiry can complement existing methods for studying creativity. When it comes to non-improvising artists, this may be especially true in terms of their psychological, emotional, and motivational approaches to creativity. It is crucial to understand how aesthetic ideals are transmitted within a performing artist and how this knowledge impacts the music performance sector, with implications for how music performance is taught to prospective musicians. With our current understanding of the music teaching and learning process, music practitioners are encouraged to stay up to date with the latest study findings as the diverse emergence of neuromusical research will undoubtedly have a substantial influence on music performance.

## Data Availability Statement

The original contributions presented in the study are included in the article/supplementary material, further inquiries can be directed to the corresponding author/s.

## Author Contributions

The author confirms being the sole contributor of this work and has approved it for publication.

## Conflict of Interest

The author declares that the research was conducted in the absence of any commercial or financial relationships that could be construed as a potential conflict of interest.

## Publisher’s Note

All claims expressed in this article are solely those of the authors and do not necessarily represent those of their affiliated organizations, or those of the publisher, the editors and the reviewers. Any product that may be evaluated in this article, or claim that may be made by its manufacturer, is not guaranteed or endorsed by the publisher.
